# Valproate-induced severe symptomatic hyponatremia in a patient with schizoaffective disorder: a case study and literature review

**DOI:** 10.1192/j.eurpsy.2023.1174

**Published:** 2023-07-19

**Authors:** K. Heflin, M. ElSayed

**Affiliations:** 1 Geisel School of Medicine at Dartmouth, Hanover; 2 Psychiatry, Dartmouth Health, Lebanon; 3 New Hampshire Hospital, Concord, United States

## Abstract

**Introduction:**

The syndrome of inappropriate antidiuretic hormone secretion (SIADH) is a serious condition associated with persistently high ADH with water retention despite sufficient vascular volume. Sodium valproate (VPA), an antiepileptic indicated to treat bipolar disorder, blocks sodium (Na) and calcium ions. Few studies have examined the association between VPA and SIADH.

**Objectives:**

This abstract has two interrelated objectives: (1) to describe a VPA-associated SIADH case study we encountered in our clinical setting; and (2) to review literature for other VPA-associated SIADH cases to illuminate associations and possible risk factors.

**Methods:**

After recording a case from clinical experiences, we completed a literature review of other cases of hyponatremia associated with VPA.

We reviewed resulting artticles from searches in PubMed and in the aggregate Dartmouth Biomedical Library indices with no date or language parameters. We then searched those articles’ bibliographies.

**Results:**

Ms. A is a 63-year-old woman with schizoaffective disorder, bipolar type, hospitalized for the resurgence of visual hallucinations (VH) of “monsters” asking her to hurt herself and others. She had been adherent to VPA (500mg twice daily) and non-adherent to prescribed Olanzapine (25mg). On Day 1 (D1), her labs were concerning for serum Na 119mEq/L (n=135-145), serum osmolality (SOsm) 264mEq/L (n=275-295), and inappropriately high urine osmolality 111mOsm/kg (n <100 mosmol/kg in hyponatremia) and urine Na 34mEq/L (n <20 mosmol/kg in hypovolemic hyponatremia). Her VPA level was 73.6 mcg/mL.

She was restarted on her home psychiatric medications for VH, and her hyponatremia responded to water restriction, with serum osmolality at 292mEq/L by D4 (see Figure). She was admitted to the inpatient psychiatric unit for concerns of persistent VH. On D13, her SOsm worsened to 267mEq/L and VPA was discontinued at that time. On D19, SOsm improved to 283mEq/L. Her VH responded well to discontinuing VPA and adding Risperidone (titrated to 6mg) and on D22 she was discharged home. Given the chronological sequence of her newly developed VH, the patient’s hallucinations were likely multifactorial, with contribution from hyponatremic encephalopathy-related psychosis.

Our literature review found ten articles reporting thirteen other cases of VPA-associated SIADH (see Table). Our patient shared demographics with most previously reported cases: being older in age and having polytherapy and a low baseline Na. None of the previous case reports showed specific drug interactions to be particularly likely causes of hyponatremia.

**Image:**

**Figure:**
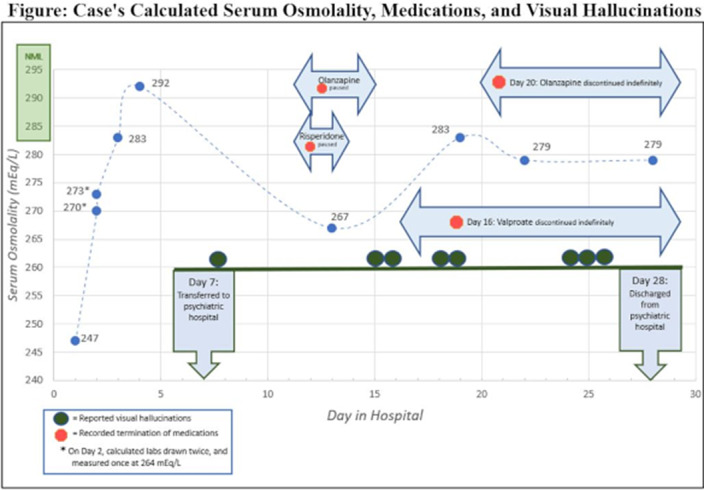

**Image 2:**

**Figure:**
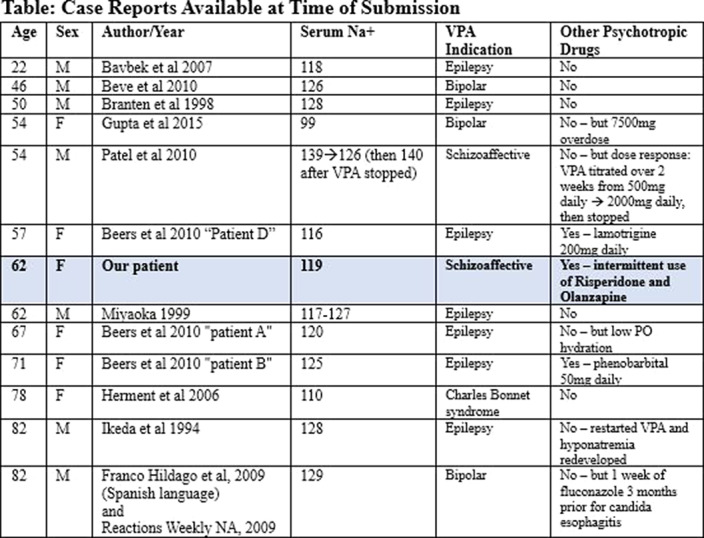

**Conclusions:**

Although VPA-associated SIADH is a rare phenomenon, caution is warranted when evaluating patients with VPA use presenting acutely with psychosis and hyponatremia. These symptoms could be the manifestation of hyponatremic encephalopathy-related psychosis.

**Disclosure of Interest:**

None Declared

